# A Comparison of the Biological Features of Prostate Cancer with (PSA+, PSMA+) Profile according to RKIP

**DOI:** 10.1155/2013/409179

**Published:** 2013-08-07

**Authors:** Awatef Ben Jemaa, Yosra Bouraoui, Sataa Sallami, Yassine Nouira, Ridha Oueslati

**Affiliations:** ^1^Unit of Immunology and Microbiology Environmental and Carcinogenesis (IMEC), Faculty of Sciences of Bizerte, University of Carthage, Zarzouna 7021, Tunisia; ^2^Department of Urology, Hospital of La Rabta, Tunis 1007, Tunisia

## Abstract

*Purpose*. To investigate differences in the biological features of the most immunoexpressed prostate cancer (PC) profiles (PSA+, PSMA+) according to the RKIP. *Methods*. 19 PC with dominant Gleason grade ≥8 were studied. Expression of PSA, PSMA, RKIP, Raf-1, MEK-1, ERK-1, ERK-2, p-Akt (T308), p-Akt (S473), NF-**κ**B p50, and NF-**κ**Bp65 were detected immunohistochemically. *Results*. Loss of RKIP in the most immunoexpressed PC (PSA+, PSMA+) profile was associated with increased levels of PSA and PSMA expression. Intensities of immunoreactions to PSA and PSMA were higher in cancer cells negative for RKIP (12.51 ± 1.6 and 34.95 ± 1.92) compared to those positive for RKIP (4.68 ± 1.11 and 28.56 ± 0.91). In parallel, missing RKIP expression in PC patients with PSA+, PSMA+ profile was connected with increased components of both Raf-1/MEK/ERK and NF-**κ**B (p65/p50), whereas Akt is activated independently of RKIP. *Conclusions*. Although characterized by the same (PSA+, PSMA+) profile, PC phenotype missing the RKIP related to invasive potential and greater biological aggressiveness reflected in overexpression of components of Raf-1/MEK/ERK and NF-**κ**B (p65/p50) in which Akt is activated independently of RKIP. Taking into account the PC phenotypes according to RKIP among PSA-PSMA profiles may improve distinguishing them from cancers that will become more aggressive and therefore adapt the therapeutic strategies in those patients.

## 1. Introduction

Prostate cancer (PC) is the most frequently diagnosed cancer among men and the second leading cause of male cancer deaths [1]. In spite of an initial sensitivity, PC cells became resistant to androgen deprivation therapy. At such stage, PC represents a transition to lethal phenotypes of the disease to which majority of the patients succumb [2]. Prostate specific antigen (PSA) and prostate specific membrane antigen (PSMA) have been identified as the most promising biomarkers in diagnosis and treatment of PC [3, 4]. Both PSA and PSMA expression levels are regulated by androgens through the androgen receptor (AR) [5]. Several signaling pathways are involved both in early tumorigenesis of PC and in androgen-refractory disease, for which no curative treatment exists currently, among them MAPKs, NF-*κ*B, and Akt pathways [6]. The terminal kinases in the Ras/Raf-1/MEK/ERK pathway are the extracellular regulated kinases (ERKs) [7]. ERK is implicated in various cellular processes, including proliferation, differentiation, apoptosis, and transformation [8]. 

It has been well known that the NF-*κ*B (p50/65) pathway plays a critical role in prostate cell development and progression of various human malignancies [9, 10]. The NF-*κ*B (p50/p65) pathway is affected by Akt [11]. PI3K/Akt pathway is a major survival pathway in the development and progression of malignant prostate [6]. Akt is activated by a dual regulatory mechanism that requires both translocation to the plasma membrane and phosphorylation at T308 and S473 [12].

By influencing the Raf kinase and NF-*κ*B pathways, RKIP is considered to play a pivotal role in the pathogenesis of PC [13, 14]. Accumulating pieces of evidences indicate that RKIP expression induces several of the characteristics generally associated with prostate cancerous growth and spread [15]. We have previously showed that PSA and PSMA are often coexpressed in human cancerous prostate [16]. At present, little is known about the biological features of this mostly immunoexpressed PC profile (PSA+, PSMA+). In this work, we initially analysed the expression of PSA and PSMA according to RKIP and then investigated the components of Ras/Raf-1/MEK/ERK, NF-*κ*B (p50/p65), and Akt (T308/S473) pathways in conjunction with PSA and PSMA according to RKIP in human cancerous prostate with (PSA+, PSMA+) profile to assess the biological characters of this mostly immunoexpressed PC profile. 

## 2. Material and Methods

### 2.1. Patients

Prostates were obtained from radical prostatectomy from 19 men (aged from 57 to 88 years) diagnosed with PC (dominant Gleason grade ≥ 8). The patients did not receive hormonal therapy before prostatectomy. All pathological, clinical, and personal data were anonymized and separated from any personal identifiers. All the procedures followed were examined and approved by the Hospital of La Rabta of Tunis and Military Hospital of Tunis (HMPIT) (Tunisia).

### 2.2. Antibodies

The primary antibodies used were mouse anti-human PSA (ER-PR8), mouse anti-human PSMA (3E6) (Dako, Glostrup, Denmark), rabbit monoclonal anti-human RKIP (Abcam plc, Cambridge, United Kingdom), polyclonal goat anti-human Raf-1, mouse anti-human MEK-1, rabbit anti-human ERK-1, mouse anti-human ERK-2 (Santa Cruz Biotechnology, CA, USA), rabbit anti-human phospho-Akt (T308), rabbit anti-human phospho-Akt (S473) (Bioworld Technology, USA), rabbit anti-human NF-*κ*Bp65, and mouse anti-human NF-*κ*Bp50 (Santa Cruz Biotechnology, CA, USA). 

### 2.3. Immunohistochemistry

For immunohistochemistry analysis, tissues were fixed 10% formaldehyde, dehydrated, and embedded in paraffin. Sections (5 m thick) were processed following the avidin-biotin-peroxidase complex (ABC) method. Following deparaffinization, sections were hydrated, incubated for 30 minutes in 0.3% H_2_O_2_ diluted in methanol to reduce endogenous activity. To retrieve the antigen, the sections were incubated with 0.1 M citrate buffer (pH 6) for 2 minutes in a conventional pressure cooker. After incubation with TBS containing 3% donkey serum, the primary antibodies were applied at a dilution of 1/50 (PSMA, Raf-1, MEK-1, ERK-1, ERK-2, NF-kBp65, and NF-kBp50), 1/300 (RKIP), 1/100 (PSA, phospho-Akt (T308) and phospho-Akt (S473)) in TBS at room temperature overnight. Afterwards, the sections were washed twice and then incubated with swine anti-rabbit (RKIP, Raf-1, ERK-1, phospho-Akt (T308), phospho-Akt (S473), and NF-kBp65) and rabbit anti-mouse (PSA, PSMA, MEK-1, ERK-2, and NFkBp50) biotinylated immunoglobulin (Dako, Barcelona, Spain) at 1 : 500 in TBS for 1 hour. The sections were incubated with a standard streptavidin-biotin complex (Vector Laboratories, Burlingame, CA, USA) at 1 : 500 and developed with 3,30-diaminobenzidine (DAB), using the glucose oxidase-DAB-nickel intensification method. Immunochemical procedure specificity was checked using negative controls (sections incubated with preimmune serum or blocking peptides; Santa Cruz Biotechnology).

A comparative histological quantification of immunolabeling among the different prostates was performed for each antibody. Of each prostate, six histological sections were selected at random. In each section, the staining intensity (optic density) per unit surface area was measured with an automatic image analyzer (Motic Images Advanced version 3.2, Motic China Group Co., China) in five light microscopic fields per section, using the ×40 objective. Delimitation of surface areas was carried out manually using the mouse of the image analyzer. For each positively immunostained section, one negative control section (the following in a series of consecutive sections) was also used, and the optic density of this control section was taken away from that of the stained section. From the average values obtained (by the automatic image analyzer) for each prostate, the means ± SD for prostatic cancer were calculated. The same results were obtained by two different observers. The number of sections examined was determined by successive approaches to obtain the minimum number required to reach the lowest SD. The statistical significance between means of the different prostate group's samples was assessed by the Fisher exact and the *t*-tests at *P* < 0.05 (GraphPad PRISM 5.0 computer program).

## 3. Results

### 3.1. Expression of PSA, PSMA, RKIP, Raf-1, MEK-1, ERK1/2, p-Akt (T308/S473), and NF-*κ*B (p50/p65) in Prostate Cancer

This study was performed in prostate cancer tissues which were evaluated for PSA, PSMA, RKIP, Raf-1, MEK-1, ERK1/2, p-Akt (T308/S473), and NF-*κ*B (p50/p65) expression by immunohistochemistry analysis using isoform-specific antibodies. 

**Figure 1:**
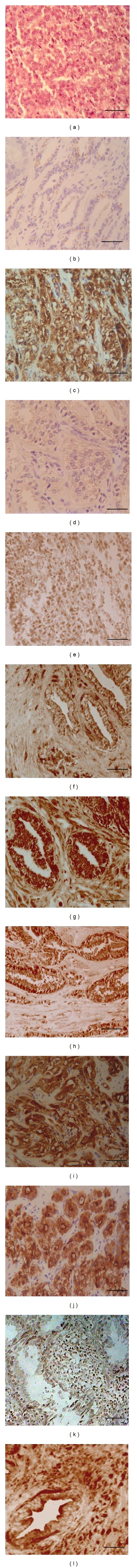
Representative human prostatic carcinomas showing immunostaining for PSA, PSMA, RKIP, Raf-1, MEK-1, ERK-1, ERK-2, p-Akt (T308), p-Akt (S473), NF-*κ*B p50, and NF-*κ*B p65. High grade prostatic carcinoma stained with hematoxylin/eosin (a). Low PSA immunoreactivity in neoplastic epithelial cells (b). Strong and diffuse cytoplasmic and membranous PSMA expression in infiltrating prostatic malignant cells (c). Immunostaining for RKIP predominantly in the cytoplasm (d). Nuclear immunostaining for Raf-1 in neoplastic epithelial cells (e). Immunostaining for MEK-1 in the nucleus and the cytoplasm (f). ERK-1 showed cytoplasmic and nuclear immunoreactions of epithelial cells (g). Immunostaining for ERK-2 in the nucleus and the cytoplasm of epithelial cells (h). Strong and diffuse p-Akt (T308) expression in cytoplasm of neoplastic acinar structure in prostatic carcinoma (i). p-Akt (S473) showed strong and diffuse cytoplasm and cytoplasmic membranes in infiltrating malignant cells (j). NF-*κ*B p50 immunoreactivity in the nucleus and the cytoplasm (k). NF-*κ*B p65 showed cytoplasmic and nuclear immunoreactions of epithelial cells in prostate cancer (l). Bar: 20 *μ*m.

As shown in Figure 1, immunoreactions to PSA, RKIP, p-Akt (T308), and p-Akt (S473) were found predominately in the cytoplasm of epithelial cells, while immunoexpression of Raf-1, MEK-1, ERK-1, ERK-2, NF-*κ*B p50, and NF-*κ*Bp65 was found in both the cytoplasm and nucleus of epithelial cells. However, immunoreactivity for PSMA appeared in both cytoplasmic and membranous pattern in neoplastic epithelial cells. 

Expression of PSA, PSMA, and RKIP was, respectively, detected in 14 (73.6%), 18 (94.7%), and 8 (42.1%) of all tumours. Raf-1, MEK-1, ERK-1, and ERK-2 were, respectively, expressed by 9 (47.3%), 10 (52.6%), 12 (63.1%), and 7 (36.8%) of all prostate carcinomas. Immunopositivity for NF-*κ*B p50 or NF-*κ*Bp65 was observed in 7 (36.8%) of patients. Moreover, 78.9% (15 of 19) of prostate cancer cases exhibited p-Akt (T308) immunoexpression, while 73.6% (14 of 19) of patients exhibited positivity to p-Akt (S473). 

### 3.2. PSA and PSMA Expression according to RKIP among (PSA+, PSMA+) Profile

**Figure 2:**
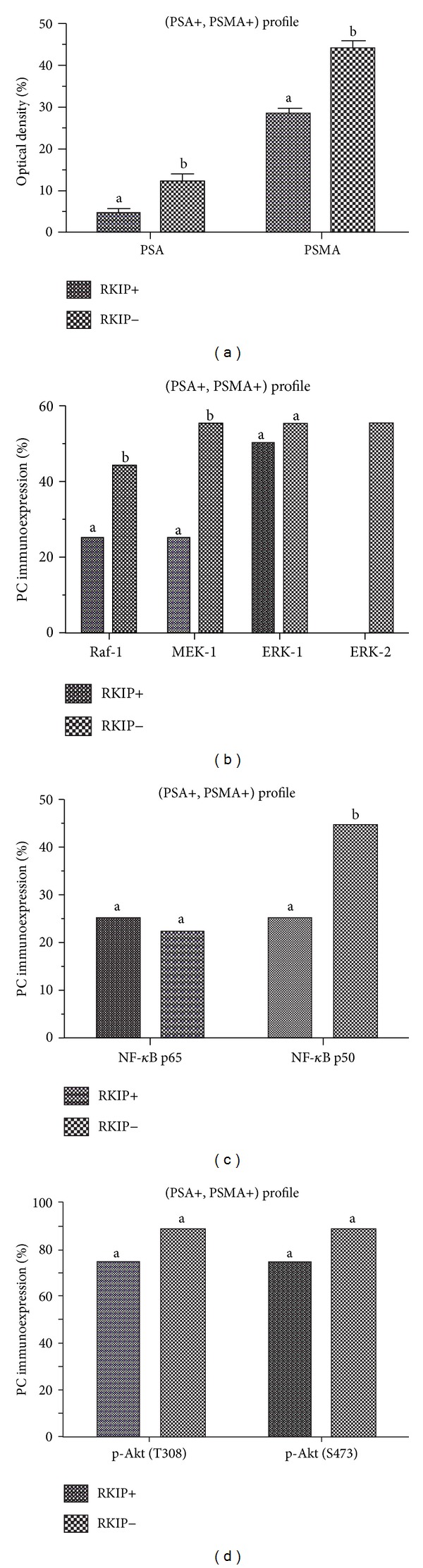
Comparison of the PSA, PSMA (a), Raf-1, MEK-1, ERK-1, ERK-2 (b), NF-*κ*B p65, NF-*κ*B p50 (c), p-Akt (T308) and p-Akt (S473) (d), expression among (PSA+, PSMA+) profile according to groups: PC patients with positive immunoreactions to RKIP and PC patients with negative immunoreactions to RKIP. Values denoted by different superscripts are significantly different from each other. Those values sharing the same superscript are not statistically different from each other. Statistical analysis refers to each antibody separately. Significance was determined at *P* < 0.05.

Figure 2(a) shows the distribution of PSA and PSMA staining intensities in prostate cancer patients either in the presence or in the absence of RKIP among (PSA+, PSMA+) profile. The intensities of immunoreactions to PSA and PSMA were significantly more intense in PC samples with negative immunoreactions to RKIP (12.51 ± 1.6 and 34.95 ± 1.92) than those with positive immunoreactions to RKIP (4.68 ± 1.11 and 28.56 ± 0.91) (*P* < 0.05). Moreover, in each PC group (first group: positive for RKIP and second group: negative for RKIP) the expression of PSMA was greater than PSA by several folds (Figure 2(a)). 

### 3.3. Raf/MEK/ERK Transduction Pathway according to RKIP among (PSA+, PSMA+) Profile

We examined the expression of each signaling molecule of Raf-1/MEK/ERK axis in two PC groups: first group represents patients with positive immunoreactions to RKIP and second group represents patients lacking of RKIP expression. As shown in Figure 2(b), the percentage of each signaling molecule of Raf-1/MEK/ERK transduction pathway positive tissues increased significantly from the group of patients positive for RKIP and those without immunoreactions to RKIP, whereas there was no significant positivity for ERK-1 between the two PC groups. Moreover, no immunoreactivity to ERK-2 was observed in PC patients with positive immunoreactions to RKIP (Figure 2(b)). 

### 3.4. NF-*κ*B (p50/p65) according to RKIP among (PSA+, PSMA+) Profile

As shown in Figure 2(c), the loss of RKIP expression was associated with significantly increased positivity for NF-*κ*B p50 subunit (25% and 44.4%, *P* < 0.05) in PC patients with (PSA+, PSMA+) profile, while there was no significant difference in the positivity for NF-*κ*B p65 subunit (25% and 22.2%). 

### 3.5. Akt Activation (p-Akt (T308) and p-Akt (S473)) according to RKIP among (PSA+, PSMA+) Profile

We compare the Akt activation either in the loss or in the presence of RKIP among (PSA+, PSMA+) profile. As shown in Figure 2(d), the percentages of p-Akt (T308) and p-Akt (S473) positive tissues were regarded. The loss of RKIP was associated with an increase of expression of each molecule but without significant differences (from 75% to 88.8% for each molecule). 

## 4. Discussion

Although several tissue microarray studies have been done on RKIP and clinical outcome, our study is the first to use RKIP for the evaluation of the biological feature of the most immunoexpressed PC (PSA+, PSMA+) profile. As a starting point, we compared the expression of PSA and PSMA according to RKIP among (PSA+, PSMA+) profile. Although our PC cases that coexpressed PSA and PSMA are mostly poorly differentiated adenocarcinoma (Gleason score ≥ 8), they reacted differently with RKIP. In fact, some of PC patients were reflected by the expression of RKIP, whereas in others it is reflected by the loss of this protein. We interpret that human PC is a complex disease characterized by considerable heterogeneity in its behavior. However, either in the presence of RKIP or in its loss, PSMA was several folds greater than PSA in each poorly differentiated adenocarcinoma group with (PSA+, PSMA+) profile. Consistent with the correlation between PSA, PSMA expression, and tumor stage, it was revealed that PSA on tissue level was inverse related to Gleason grade, whereas increased levels of PSMA are associated with high-grade prostate cancers [17, 18]. Interestingly, loss of RKIP expression was associated with increased levels of both PSA and PSMA expression. Thus, we suggested an inverse association between RKIP and PSA-PSMA expression in prostatic adenocarcinoma patients. Although PSA and PSMA have been reported to be expressed in reciprocal manner in benign prostatic hyperplasia and prostate carcinomas, their expression are maintained upon PC progression [16]. However, loss of RKIP may be considered to be a marker of PC. Moreover, inhibition of RKIP expression makes certain prostate cells more metastatic [19, 20]. RKIP is not thought to alter the tumorigenic properties of PC cells, rather it is thought to be a suppressor of metastasis and may function by decreasing vascular invasion [20]. Inversely to RKIP, PSA and PSMA have been shown to be prospective markers to detect PC micrometastasis [21]. Our results showed that missing of RKIP expression in PC patients with (PSA+, PSMA+) profile was associated with increase in the positivity of each signaling molecule Raf-1, MEK-1, ERK-1, and ERK-2. In this paper, we detected the nonphosphorylated form of RKIP that negatively regulates the Raf/MEK/ERK pathway by interfering with the activity of Raf-1 [15]. Various isoforms of PKC have been shown to phosphorylate RKIP on S153 which results in the disassociation of Raf-1 and RKIP. For this reason, we thought that missing of RKIP in its nonphosphorylated form in some PC patients may be due to its conversion to the phosphorylated state by PKC which subsequently stimulate both the Raf/MEK/ERK and of G-protein coupled receptors pathways [14, 15]. Since the loss of RKIP was concomitant with increased expression of PSA, PSMA, and Raf-1/MEK/ERK signaling pathway, we suggested a cross-talk between RKIP/Raf-1/MEK/ERK cascade, PSA and PSMA expression in PC. This cross-talk may contribute to the aggressiveness and metastasis of PC in patients that the disease status is reflected by the loss of RKIP. In support of this, in LNCaP and PC3-PSMA cells, overexpression of MAPKs (ERK1/2 and p38) mediated by bFGF, fundamental agent of angiogenesis upregulates PSMA expression [22]. In addition, Ras/MEK/ERK signaling can contribute to the stimulation of PSA expression and sustain the growth of androgen-dependent and androgen-independent PC cells [23]. 

In another hand, in the present study we demonstrated that the loss of RKIP expression was also associated with increased expression of p65 and p50 NF-*κ*B subunits in PC patients with (PSA+, PSMA+) profile. Interacting in a similar fashion with Raf-1 and MEK, RKIP was previously found to associate with NIK and TAK1 which are the upstream kinases of NF-*κ*B pathway and to inhibit their activation [14, 24]. Thus, our results could suggest a synergistic effect between RKIP/ERK and RKIP/NF-*κ*B signaling on PSMA and PSA expression. It is well established that ERK1/2 has a role in the process of activation of NF-*κ*B transcription factor [25]. We have previously showed that NF-*κ*B activation could increase PSA production in primary PC cases compared to BPH [10]. However, experimental data demonstrated that NF-*κ*B expression is in turn enhanced following PSMA stimulation in LNCaP cells [25].

In a final approach, we investigated the cross-talk between RKIP and Akt activation (T308/S473) among PC patients with (PSA+, PSMA+) profile. In the present paper, RKIP does not seem to be directly implicated in the activation of Akt in PC patients with (PSA+, PSMA+) profile. In fact, we did not find a significant increase in the expression of both p-Akt (T308) and p-Akt (S473) between PC patients with positive immunoreactions to RKIP and those missing the RKIP expression. However, these results do not exclude the involvement of Akt pathway in the regulation of PSA and PSMA expression without bypass by RKIP in the PC patients with the above profile.

As endowed with signaling activity in prostate cancer cells, PSMA has profound influence on the survival, proliferation, and migration of prostate tumor cells. Furthermore, PSMA overexpression in prostate cancer patients is related to a worse prognosis [25, 26]. In respect to our study, these observations indicate that overexpression of PSMA in PC patients lacking RKIP expression may provide to these patients a feature of potential aggressiveness compared to those with positive RKIP expression.

**Figure 3:**
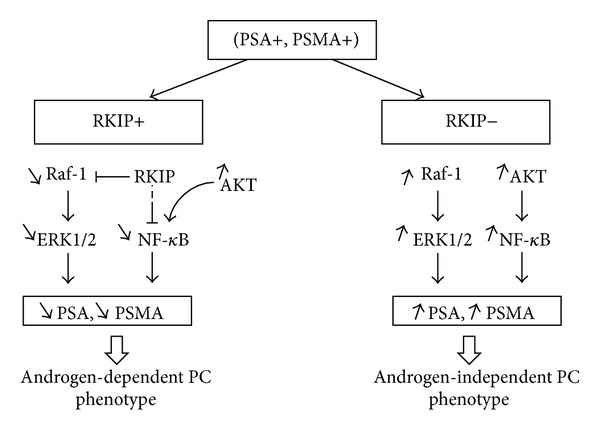
Comparison of the biological features of prostate cancer with (PSA+, PSMA+) profile according to RKIP. Among (PSA+, PSMA+) profile, loss of RKIP leads to upregulation of Raf-1/ERK and NF-*κ*B pathways which subsequently stimulate PSA and PSMA expression, whereas Akt is activated independently to RKIP. According to RKIP, PC patients with (PSA+, PSMA+) profile could exhibit the feature of two different PC phenotypes: an androgen-dependent phenotype for PC patients keeping the RKIP and an androgen-independent phenotype for those missing the RKIP.

Several investigations have indicated that various growth factors and cytokines stimulate the AR through the Raf-1/MEK/ERK, NF-*κ*B, and Akt pathways at low level or absent in the androgens [27]. In this state of the disease, patients are on androgen deprivation and ultimately progress to castrate-resistant PC, for which no curative treatment exists currently [28, 29]. Although each pathway is conceptually linear, Raf-1/MEK/ERK, NF-*κ*B, and Akt pathways are often coordinately deregulated toward hormone-refractory PC and contribute to their more malignant or aggressive phenotype [30]. Raf-1/MEK/ERK, NF-*κ*B, and Akt signaling is known to induce together potent antiapoptotic effects, which enhance tumorigenesis of PC and contribute to PC cell survival following androgen withdrawal [27]. Moreover, androgens could regulate the expression of PSA and PSMA genes through the binding of the AR to the androgen responsive elements (AREs) in their promoter [5]. Therefore, the fluctuation in the expression of PSA and PSMA between poorly differentiated PC cases that expressed the RKIP and those lacking this molecule may be connected to the level of AR and/or androgen present in the tumor microenvironment. In support of this, it was previously reported that the heterogeneity in the expression of the AR increases with increasing Gleason score [31]. Furthermore, Denmeade et al. discovered that inversely to PSA, PSMA activity in prostate cancer cell lines increased as cells became more androgen independent [5]. Thus, these observations and our results suggested that progression of PC to hormone-refractory phenotype may in part be due to the loss of RKIP leading to upregulation of Raf-1/ERK and NF-*κ*B pathways which subsequently stimulate PSA and PSMA expression, whereas Akt is activated independently to RKIP (Figure 3). 

On the basis of the above results, it seems that according to RKIP, our PC patients with (PSA+, PSMA+) profile could exhibit the feature of two different PC phenotypes: an androgen-dependent phenotype for PC patients keeping the RKIP and an androgen-independent phenotype for those missing the RKIP (Figure 3). Consequently, our study supports the heterogeneity and the complex clonal of PC disease [32]. It is possible that this later PC phenotype might reflect subpopulation of prostate tumor that will eventually escape hormonal control and relapse to androgen-independent state that is basically lethal. 

Taken together, despite being homogenously poorly differentiated adenocarcinomas, according to RKIP, the most immunoexpressed PC profile (PSA+, PSMA+) exhibited two heterogeneous PC phenotypes with distinct biological features. Significant attention should be given to the PC phenotypes on the basis of RKIP among PSA-PSMA profiles which may lead to distinguishing them from cancers that will become more aggressive and therefore adapt the therapeutic strategies in those patients.
